# Eotaxin-3 as a Plasma Biomarker for Mucosal Eosinophil Infiltration in Chronic Rhinosinusitis

**DOI:** 10.3389/fimmu.2019.00074

**Published:** 2019-02-04

**Authors:** Takechiyo Yamada, Yui Miyabe, Shigeharu Ueki, Shigeharu Fujieda, Takahiro Tokunaga, Masafumi Sakashita, Yukinori Kato, Takahiro Ninomiya, Yohei Kawasaki, Shinsuke Suzuki, Hidekazu Saito

**Affiliations:** ^1^Department of Otorhinolaryngology, Head and Neck Surgery, Graduate School of Medicine, Akita University, Akita, Japan; ^2^Clinical Laboratory Medicine, Department of General Internal Medicine, Graduate School of Medicine, Akita University, Akita, Japan; ^3^Department of Otorhinolaryngology-Head and Neck Surgery, Faculty of Medical Science, University of Fukui, Fukui, Japan

**Keywords:** eotaxin-3, plasma biomarker, eosinophil, nasal polyp, rhinosinusitis

## Abstract

**Objective:** Chronic rhinosinusitis with nasal polyps exhibits marked eosinophilic infiltration and its mucosal eosinophilia is associated with more severe symptoms. The Japanese epidemiological survey of refractory eosinophilic chronic rhinosinusitis found that patients with nasal polyps required multiple surgeries when there were higher infiltrating eosinophils in the mucosa. In order to identify plasma biomarkers for local eosinophil infiltration in rhinosinusitis for surgery, we examined the levels of molecules in the plasma of patients and compared the number of infiltrating eosinophils in the nasal mucosa.

**Materials and Methods:** Mucosal tissues from 97 patients with chronic rhinosinusitis (CRS) were obtained from the nasal polyps during surgery. Tissues were immediately fixed and sections were stained with hematoxylin-eosin. The number of eosinophils in the mucosa was counted at HPF (x 400). Blood samples were obtained and the plasma was stored at −80°C. We measured the plasma cytokine and chemokine levels using multiple assay systems according to the manufacturers' protocols. The tissues were divided into high- and low-eosinophil mucosal infiltration group for recurrence after endoscopic sinus surgery (ESS). We also observed chemokine secretion from nasal fibroblasts.

**Results:** The plasma level of eotaxin-3/ CC chemokine ligand 26 (CCL26) was significantly higher in the high-eosinophil mucosal infiltration group (*p* < 0.005). The number of infiltrating eosinophils in the mucosa was significantly higher in the group with the higher eotaxin-3 level (*p* < 0.001), but there was no significant difference in the blood eosinophil numbers among two groups. A significant positive correlation was found between the mucosal eosinophil count and the plasma levels of eotaxin-3 (*p* < 0.005). The levels of interleukin 33 (IL-33) (*p* < 0.001) and thymic stromal-derived lymphopoietin (TSLP) (*p* < 0.005) were significantly higher in the high-level eotaxin-3 group. IL-13 strongly induced the secretion of eotaxin-3 from human nasal fibroblasts (*p* < 0.05).

**Conclusion:** This is the first report suggesting eotaxin-3 as a plasma biomarker for mucosal eosinophil infiltration. Furthermore, the level of eotaxin-3 was found to be closely related to IL-33 and TSLP levels which indicate respiratory diseases.

## Introduction

Chronic rhinosinusitis demonstrates marked heterogeneity, both at the molecular pathophysiological level and at the clinical phenotype level. This disease is divided into 2 subgroups, chronic rhinosinusitis with nasal polyps (CRSwNP) and chronic rhinosinusitis without nasal polyps (CRSsNP) (1). Nasal polyps have negative effects on many aspects of the quality of life (physical health, general health, social functioning, sleep, mental health, and workplace absenteeism) due to nasal obstruction, olfactory disturbance, rhinorrhea, and symptoms caused by lower airway involvement (2, 3). In patients 80–90% of the nasal polyps are characterized by prominent eosinophilia (4). The clinical classification of CRSwNP according to the degree of eosinophilic infiltration in nasal polyps is controversial (5).

Eosinophilic chronic rhinosinusitis (ECRS) is an emerging classification of CRS, and is thought to more accurately reflect the underlying pathophysiology. There is a wide variation among reports, and no consensus currently exists regarding the cut off for the diagnosis of ECRS. Mucosal eosinophilia is defined as >10 eosinophils per high-power field (HPF) according to histopathology profiling and remodeling changes (6–8). On the other hand, mucosal eosinophilia defined as ≥70, >100, or >120 mucosal eosinophils/HPF (9–11) is associated with poorer outcomes after endoscopic sinus surgery (ESS). A study conducted by 15 institutions participating in the Japanese epidemiological survey of refractory eosinophilic chronic rhinosinusitis revealed that the cut-off value of 70 mucosal eosinophils/HPF led to the most significant difference (*P* < 0.001) in the risk of recurrence in 1,716 patients treated by ESS (12).

T helper 1 (T_H_1) cells in patients with CRSsNP and T_H_2 cells in patients with CRSwNP are dominant (13, 14). In nasal polyps, immunoreactivity of the chemokine ligand (CCL) eotaxin subfamily comprising eotaxin-1 (CCL11), eotaxin-2 (CCL24), and eotaxin-3 (CCL26) was noted (15). Staphylococcus aureus enterotoxin B stimulation of dispersed nasal polyp cells induced significant interleukin 17A (IL-17A) synthesis (16). Thymic stromal-derived lymphopoietin (TSLP) was significantly increased in eosinophilic CRSwNP, and the expression of IL-33 was enhanced in epithelial cells in both eosinophilic and non-eosinophilic CRSwNP compared with controls (17).

The nasal mucosal eosinophilic status provides prognostic information about disease severity and outcome of CRS including surgeries. In this study, we examined different molecules in order to identify a plasma biomarker for mucosal eosinophil infiltration in CRS patients with low- and high-risk requiring multiple surgeries, as well as the correlation between the nasal tissue eosinophil count and cytokine levels. The patients could be divided into two groups according to the molecular levels. Furthermore, the patients were divided into two groups according to the plasma levels, and mucosal eosinophils, blood eosinophils, and levels of other cytokines were evaluated. We also observed chemokine secretion from nasal polyp-derived fibroblasts.

## Materials and Methods

### Subjects

We assessed patients with CRS treated by ESS. The diagnosis of sinus disease was based on patient history, clinical examination, and nasal endoscopy according to the guidelines of the European Position Paper on Rhinosinusitis and Nasal Polyps (18). Our study excluded patients who received systemic or topical corticosteroids before surgery, patients whose information on systemic or topical corticosteroids was unknown, patients who were followed up for <28 days after surgery, patients whose white blood cell count was 10,000/μl or more, and patients from whom there was no pathological specimen. Preoperative demographics and medical history including sex, age, age of onset, reaction to drugs, smoking history, complications, and drug allergies, were obtained for each patient. Blood samples were taken to perform complete blood counts. This study was approved by the ethics committee of each institution including the general public through the Division of Otorhinolaryngology, Head & Neck Surgery, University of Fukui. Nasal polyps were obtained from patients with CRS.

### Histological Analysis

Mucosal tissues from patients with CRS were obtained from the nasal polyps or polypoid lesions of the ethmoid cavity during surgery. Tissues were immediately fixed in 10% formalin, embedded in paraffin, and cut into thin sections. Sections were stained with hematoxylin–eosin. The number of eosinophils in the mucosa was counted in HPF in the three densest areas with cellular infiltrate beneath the epithelial surface, and the mean number of eosinophils was calculated. Histological examinations were performed by three expert doctors blinded to the clinical data.

### Human Nasal Polyp-Derived Fibroblast Cell Culture

Nasal polyp was obtained from patients with CRS during nasal surgery. Nasal specimens were cultured in 10 cm dishes containing RPMI 1640 medium (Nissui Pharmaceutical, Tokyo, Japan) supplemented with 10% heat-inactivated FCS (Gibco, Grand Island, NY), 0.29 mg/ml glutamine, 100 U/ml penicillin, and 100 μg/ml streptomycin, at 37°C in 5 % CO_2_ and humidified air. Nasal fragments were removed and the first passage was performed. After a period of 3–4 weeks, nasal mucosa-derived fibroblast cell lines were established. The cells were used at passage numbers 3–5. Epithelial cells were confirmed not to be contaminated by immunohistochemical examination using cytokeratin and vimentin markers. The cells were cultured in the presence of IL-13 for appropriate periods, then culture supernatants were harvested and stored at −80°C.

### Enzyme-Linked Immunosorbent Assay (ELISA)

Blood samples were obtained and centrifuged immediately to prevent the degradation of complement components, and the plasma was stored at −80°C within 1 h of blood collection. We measured the plasma levels of cytokines and the culture supernatants. We conducted multiple the assay system using Multiplex Assays (Millipore, Billerica, MA) according to the manufacturers' protocols.

### Statistical Analysis

The non-parametric Mann**-**Whitney test was performed to evaluate differences. Correlation analysis was carried out using Spearman's rank correlation coefficient. We used a ROC (receiver operating characteristic) curve to calculate the area under the curve, and the closest point to top-left of ROC was determined as the optimal cut-off point. A *p*-value of < 0.05 was considered statistically significant.

## Results

### The Cytokine Levels in Low- and High- Eosinophil Mucosal Infiltration

**Figure 1:**
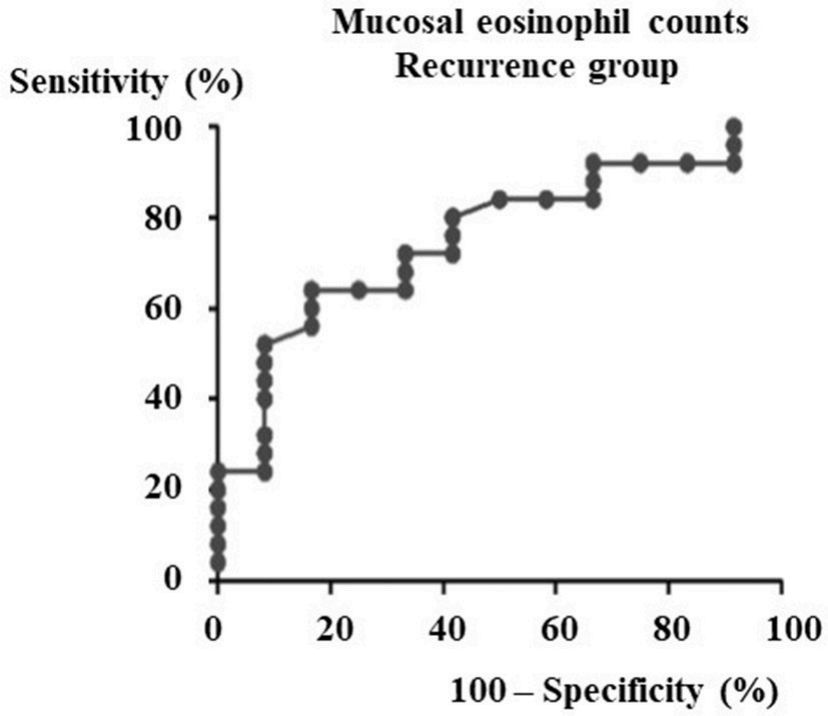
ROC curve for recurrence group after ESS to determine the cut-off point of mucosal eosinophils number. From the data of the mucosal eosinophils number diagnostic test by ROC curve against the recurrence group after ESS (Area under the curve = 0.7533, *p* = 0.014), the cut-off level of mucosal eosinophils number is 55.0 pg/ml.

A total of 67 males and 30 females aged 19 to 73 years old were included in the study. In the 37 patients that could be observed more than 3 years after surgeries, the ROC (receiver operating characteristic) curve for nasal mucosal eosinophil counts was used to discriminate the patients with recurrence from those without recurrence. Area under the curve (AUC) was 0.7533 (*p* = 0.014) (Figure 1). The optimal cut-off point of mucosal eosinophil counts was 55.0 infiltrating eosinophils/HPF to differentiate the subjects with recurrence from those without recurrence (with 64.0% sensitivity and 83.3% specificity). The high-mucosal eosinophil infiltration group was defined as those having 55.0 or more infiltrating eosinophils/HPF in the mucosa. All others were in the low-risk group. Subject characteristics are presented in Table 1. In the low- (*n* = 54) and high- eosinophil mucosal infiltration (*n* = 43), the age (mean ± SE) was 52.0 ± 1.9 and 49.5 ± 2.2 years old, the mucosal eosinophil count (/HPF) was 26.2 ± 2.2 and 134.2 ± 14.1 (*p* < 0.0001), the blood eosinophils (%) were 5.0 ± 0.6 and 7.9 ± 0.7 (*p* < 0.0005), the blood neutrophils (%) was 57.6 ± 1.3 and 54.4 ± 1.6, respectively. We next assessed the levels of eotaxin-1, eotaxin-2, eotaxin-3, IL-4, IL-5, IL-10, IL-12, IL-13, IL-21, IL-33, TSLP, TNFα, INFγ, IP-10, and MCP-1 in both groups. As shown in Table 2, the level of eotaxin-3 (84.3 ± 5.3) in the low-risk group was significantly lower than that (1122.6 ± 15.8) in the high-risk group (*p* < 0.005). There was no significant difference in the levels of other molecules including eotaxin-1, etaxin-2, or other cytokines, between the two groups in this study.

**Table 1:** Demographics by eosinophil mucosal infiltration status for chronic rhinosinusitis groups.

	**Low-eosinophil mucosal infiltration (*n* = 54)**	**High-eosinophil mucosal infiltration (*n* = 43)**
Age (y) (Mean ± SE)	52.0 ± 1.9	49.5 ± 2.2
Male, no./total (%)	36/54 (66.7)	31143 (72.1)
Mucosal eosinophil counts (/HPF)	26.2 ± 2.2	134.2 ± 14.1***
Blood eosinophil (%)	5.0 ± 0.6	7.9 ± 0.7**
Blood neutrophil (%) (Mean ± SE)	57.6 ± 1.3	54.4 + 1.6
Recurrence (%)	11.1% (2/18)	52.6% (10/19)*

High-eosinophil mucosal infiltration group is defined when the numbers of infiltrating eosinophils are 55.0 or more than 55.0 / high-power field in the mucosa. The other is low-group. P-values for comparison between low- and high- eosinophil mucosal infiltration groups. The recunence rate is shown in the patients that could be observed more than 3 years after surgeries (

* *p < 0.01*,

** *p < 0.0005*,

*** *p < 0.0001*,

*ESS, endoscopic sinus surgery; HPF, per high-powered field)*.

**Table 2:** The plasma cytokine levels of low- and high-eosinophil mucosal infiltration groups.

	**Low-eosinophil mucosa l infiltration (*n* = 54) (Mean ± SE)**	**High-eosinophil mucosal infiltration (*n* = 43) (Mean ± SE)**	***p*-value**
Eotaxin-1 (ng/ml)	1.3 ± 0.1	1.1 ± 0.1	0.369
Eotaxin-2 (ng/ml)	7.0 ± 0.4	6.0 ± 0.4	0.091
Eotaxin-3 (pg/ml)**	84.3 ± 5.3	122.6 ± 15.8	0.002**
IL-4 (pg/ml)	164.8 ± 20.6	194.0 ± 33.4	0.974
IL-5 (pg/ml)	132.9 ± 108.7	30.6 ± 10.9	0.918
IL-10 (pg/ml)	156.8 ± 118.5	139.1 ± 107.3	0.318
IL-12 (pg/ml)	56.4 ± 30.3	22.6 ± 5.0	0.305
IL-13 (pg/ml)	138.5 ± 109.2	52.0 ± 34.1	0.139
IL-21 (pg/ml)	26.8 ± 2.4	28.0 ± 2.8	0.476
IL-33 (pg/ml)	57.6 ± 18.4	165.2 ± 55.4	0.768
TSLP (pg/ml)	57.5 ± 8.5	82.6 ± 17.3	0.510
TNFa (pg/ml)	59.4 ± 6.5	51.2 ± 4.1	0.633
INFy (pg/ml)	26.2 ± 7.2	26.5 ± 3.2	0.933
IP-10 (pg/ml)	843.6 ± 118.3	716.8 ± 89.3	0.645
MCP-1 (ng/ml)	5.3 ± 0.4	5.2 ± 0.4	0.965

p-values for comparison between low- and high- eosinophil mucosal infiltration groups (

** *p < 0.005)*.

### Correlation Between Nasal Mucosal Eosinophil Count and the Plasma Cytokine Levels

A plasma biomarker should be a significant relationship to mucosal eosinophil counts in CRS. The correlation coefficients (γ) and *p-*value for comparison between mucosal eosinophil counts and the levels of eotaxin-1, eotaxin-2, eotaxin-3, IL-4, IL-5, IL-10, IL-12, IL-13, IL-21, IL-33, TSLP, TNFα, INFγ, IP-10, and MCP-1 were examined using the Spearman's rank correlation coefficient. As shown in Table 3, a significant positive correlation was found between the mucosal eosinophil count and the plasma level of eotaxin-3 (*p* < 0.005). We found no correlation between the mucosal eosinophil count and the levels of eotaxin-1, eotaxin-2, IL-4, IL-5, IL-10, IL-12, IL-13, IL-21, IL-33, TSLP, TNFα, INFγ, IP-10, or MCP-1.

**Table 3:** Correlation between nasal mucosal eosinophil counts and the plasma cytokine levels.

	**γ**	***P*-value**
Eotaxin-1	−0.113	0.267
Eotaxin-2	−0.172	0.091
Eotaxin-3	0.330	0.001**
IL-4	−0.065	0.525
IL-5	−0.007	0.944
IL-10	−0.038	0.708
IL-12	−0.148	0.147
IL-13	−0.186	0.069
IL-21	0.148	0.148
IL-33	0.041	0.691
TSLP	0.091	0.372
TNFα	−0.149	0.148
INFγ	0.111	0.273
IP-10	−0.073	0.474
MCP-1	0.029	0.778

Correlation coefficients (γ) and P-value for comparison between mucosal eosinophil counts and the plasma cytokine levels were examined using the Speannan's conelation coefficient by rank. (

** *p < 0.005)*.

### Tissue Eosinophil Infiltration and Blood Eosinophils Between High- and Low-Level Eotaxin-3 Groups

**Figure 2:**
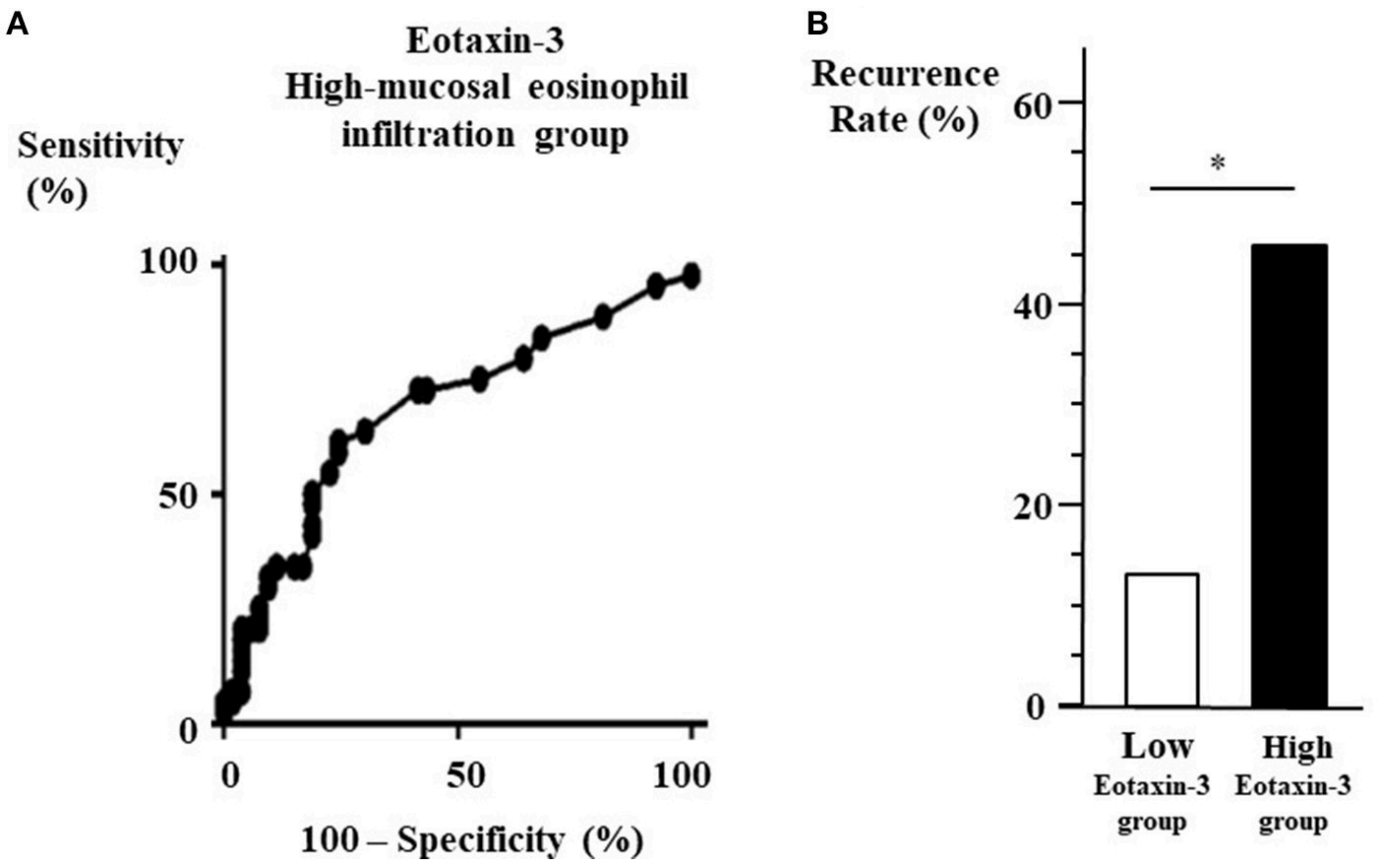
**(A)** ROC curve for high-eosinophil mucosal infiltration group to determine the cut-off point of plasma eotaxin-3 level. From the data of the eotaxin-3 diagnostic test by ROC curve against the high-eosinophil mucosal infiltration group after ESS (Area under the curve = 0.6829, *p* = 0.002), the cut-off level of eotaxin-3 is 78.8 pg/ml. **(B)** Recurrence rate between high- and low-level eotaxin-3 groups. The 37 patients that could be observed more than 3 years after surgeries were divided into two groups according to the plasma level of eotaxin-3. The recurrence rate in high-level eotaxin-3 group was higher than that in the other group (*p < 0.05).

The ROC curve for plasma eotaxin-3 levels was used to discriminate high-eosinophil mucosal infiltration group after ESS from the other group. The optimal cut-off point of plasma eotaxin-3 level was 78.8 pg/ml (with 61.4% sensitivity and 75.5% specificity) (Figure 2A). In the patients that could be observed more than 3 years after surgeries, the recurrence rate of low-level eotaxin-3 group (13.3 %) was significantly lower than that of high-level eotaxin-3 group (45.5 %) (*p* < 0.05) (Figure 2B).

**Figure 3:**
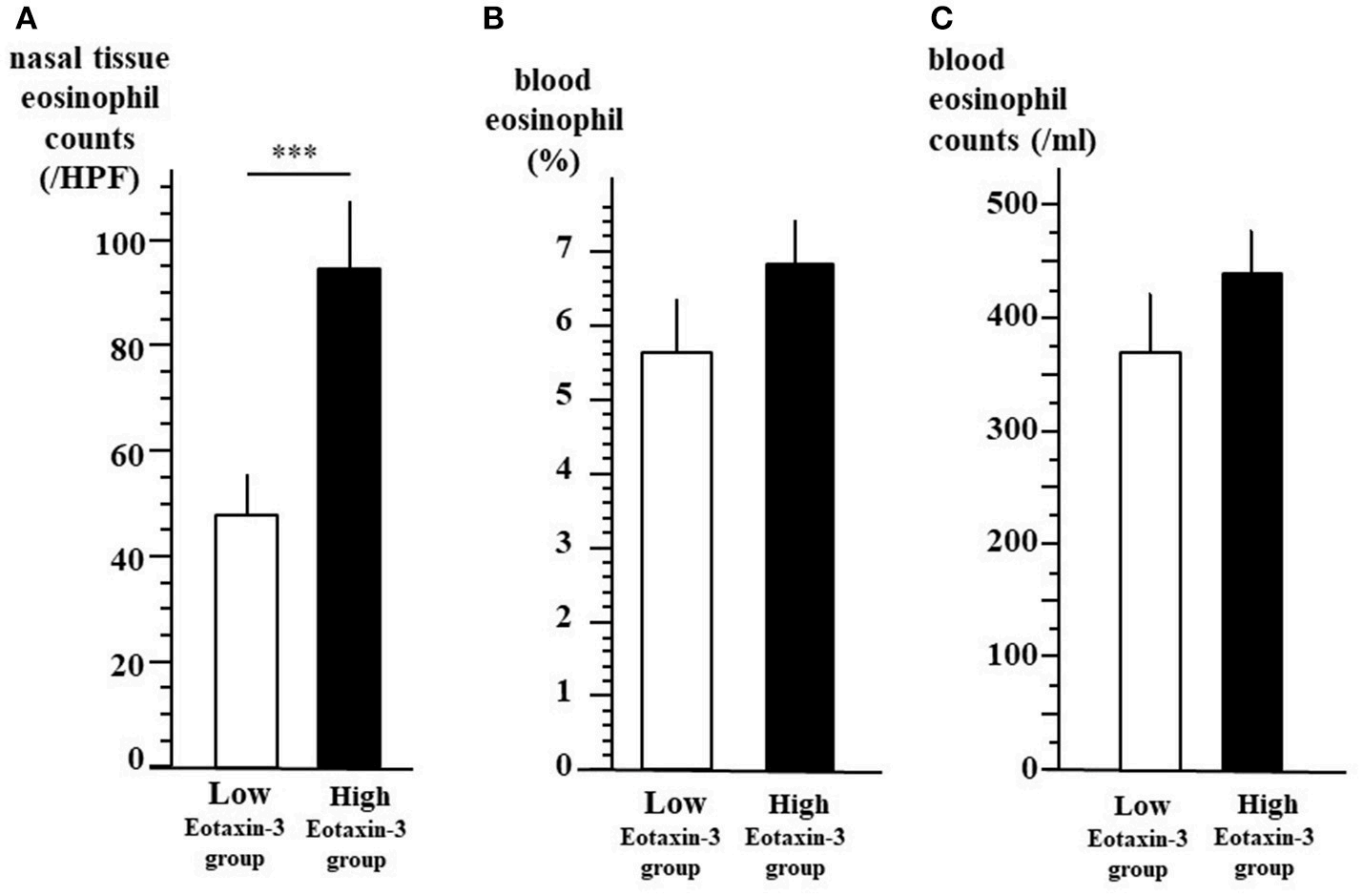
Tissue eosinophil infiltration and blood eosinophils between high- and low-level eotaxin-3 groups. All the 97 patients were divided into two groups according to the plasma level of eotaxin-3. In low-level eotaxin-3 group, the level of eotaxin-3 was 78.8 pg/ml or lower. In the high-level eotaxin-3 group, the level of eotaxin-3 were higher than 78.8 pg/ml. The mean ± SE of the nasal mucosal eosinophil count per HPF **(A)**, the percentage **(B)**, and the number **(C)** of blood eosinophil are indicated by open (low-level eotaxin-3 group) and closed (high-level eotaxin-3 group) bars and lines (***p < 0.001).

As shown in Figure 3, we divided the enrolled patients into two groups according to the plasma level of eotaxin-3. In low-level eotaxin-3 group (*n* = 42), the level of eotaxin-3 was 78.8 pg/ml or lower, whereas in the high-level eotaxin-3 group (*n* = 55), the level was higher than 78.8 pg/ml. The nasal mucosal eosinophil count (mean ± SE = 47.5 ± 7.9 / HPF) was significantly lower in low-level eotaxin-3 group than in high-level eotaxin-3 group (mean ± SE = 94.4 ± 12.9/HPF) (*p* < 0.001) (A). On the other hand, there was no significant difference in the percentage (B) or the number (C) of blood eosinophil between the two groups (low-level eotaxin-3 group: mean ± SE = 5.6 ± 0.7%, 369.4 ± 42.4/ml) (high-level eotaxin-3 group: mean ± SE = 6.8 ± 0.6 %, 433.6 ± 36.2/ml).

### Cytokine Levels Among High- and Low-Level Eotaxin-3 Groups

**Figure 4:**
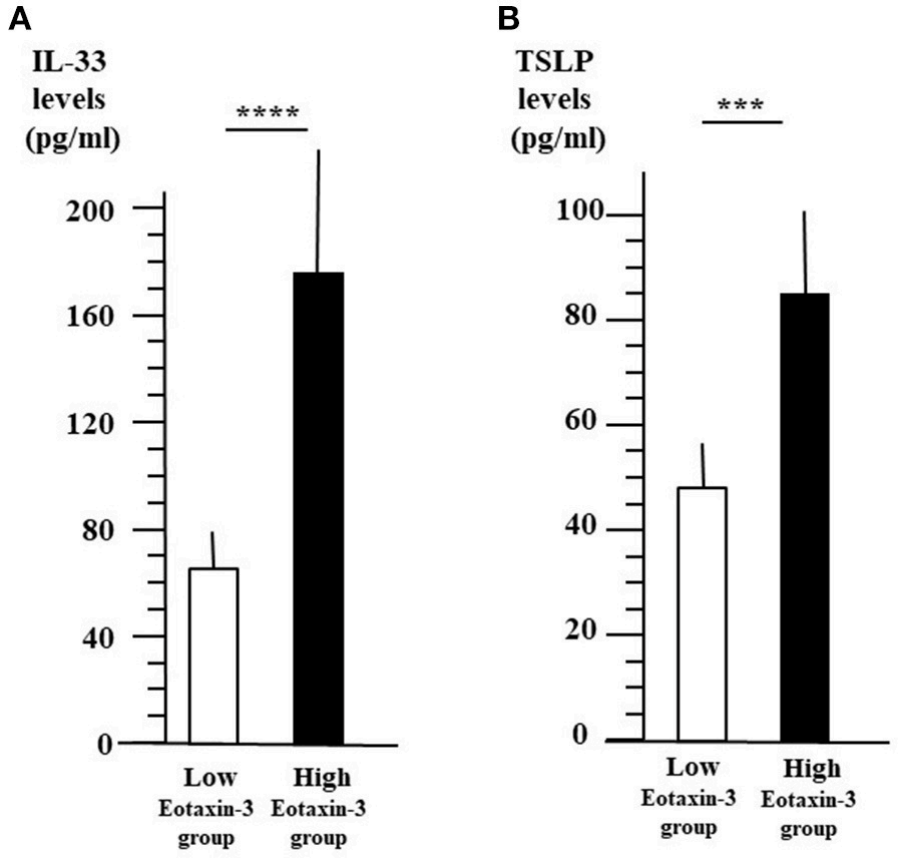
IL-33 and TSLP levels between high- and low-level eotaxin-3 groups. The patients were divided into two groups according to the plasma level of eotaxin-3 as in Figure 2. The mean ± SE of plasma IL-33 **(A)** and TSLP levels **(B)** are indicated with the open (low-level eotaxin-3 group) and closed (high-level eotaxin-3 group) bars and lines (****p < 0.001, ***p < 0.005).

We also compared the plasma levels of other cytokines between two groups according to the level of eotaxin-3 as in Figure 3. Figure 4A shows that the level of IL-33 (mean ± SE = 175.0 ± 44.4 pg/ml) was significantly higher in high-level eotaxin-3 group than that (mean ± SE = 65.6 ± 17.0 pg/ml) in low-level eotaxin-3 group (*p* < 0.001). The level of TSLP (mean ± SE = 84.8 ± 14.5 pg/ml) was also significantly higher in high-level eotaxin-3 group than those (mean ± SE = 47.4 ± 7.9 pg/ml) in low-level eotaxin-3 group (*p* < 0.005) (Figure 4B).

### IL-13-Induced Eotaxin-3 Protein Secretion in Human Nasal Polyp Fibroblasts

**Figure 5:**
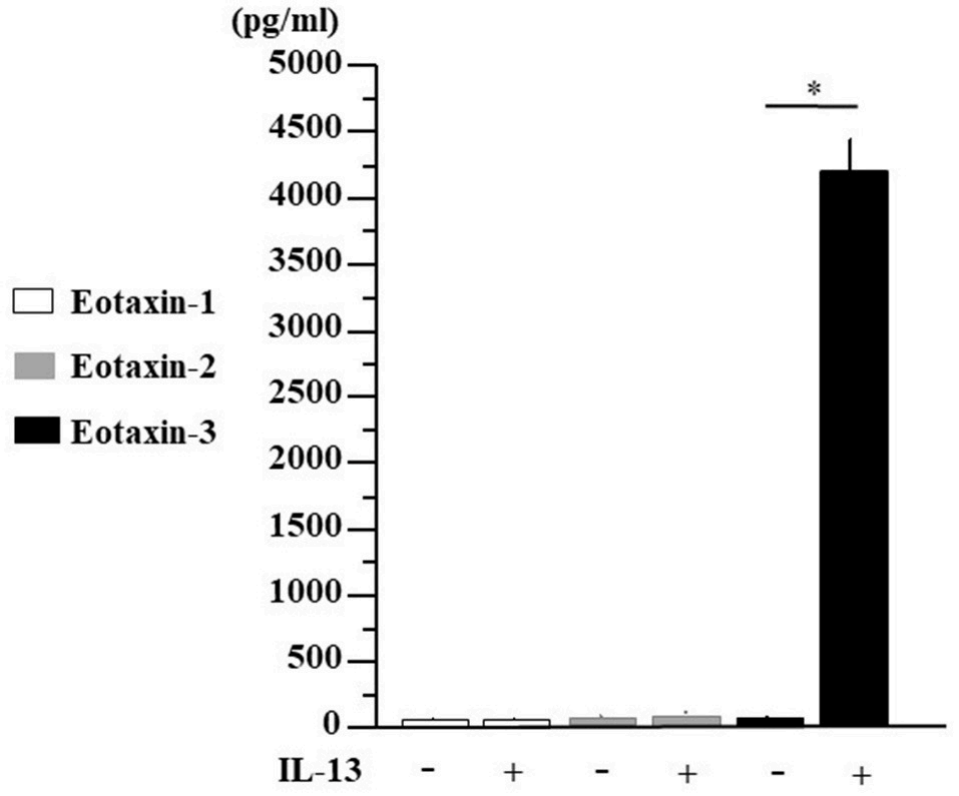
IL-13-induced eotaxin-3 protein secretion in human nasal fibroblasts. Nasal polyp was obtained from patients during surgery and fibroblast lines were established. Human nasal fibroblasts were stimulated with IL-13 (10 ng/ml) for 48 h. Supernatants were harvested for the analysis for eotaxin-1, eotaxin-2, and eotaxin-3 production by ELISA. The results are expressed as the mean ± SE (*n* = 8) *p < 0.05.

TSLP and IL-33 are closely related to innate lymphoid cells (ILCs) and T_H_2 cells, inducing IL-4, IL-5, and IL-13 production. Since the expression of eotaxin-3 in human nasal fibroblast is quite unknown, we established fibroblast lines from small pieces of human nasal polyps respectively, from 8 individuals and then examined eotaxin-3 protein secretion in fibroblasts stimulated by IL-13 for 48 h. As shown in Figure 5, IL-13 significantly enhanced the secretion of eotaxin-3 in human nasal fibroblasts (*p* < 0.05), while we could not detect any increase of eotaxin-1 or eotaxin-2. The eotaxin-3 production in the presence of IL-13 was strongly induced more than 240 times higher than in the absence of IL-13.

## Discussion

**Figure 6:**
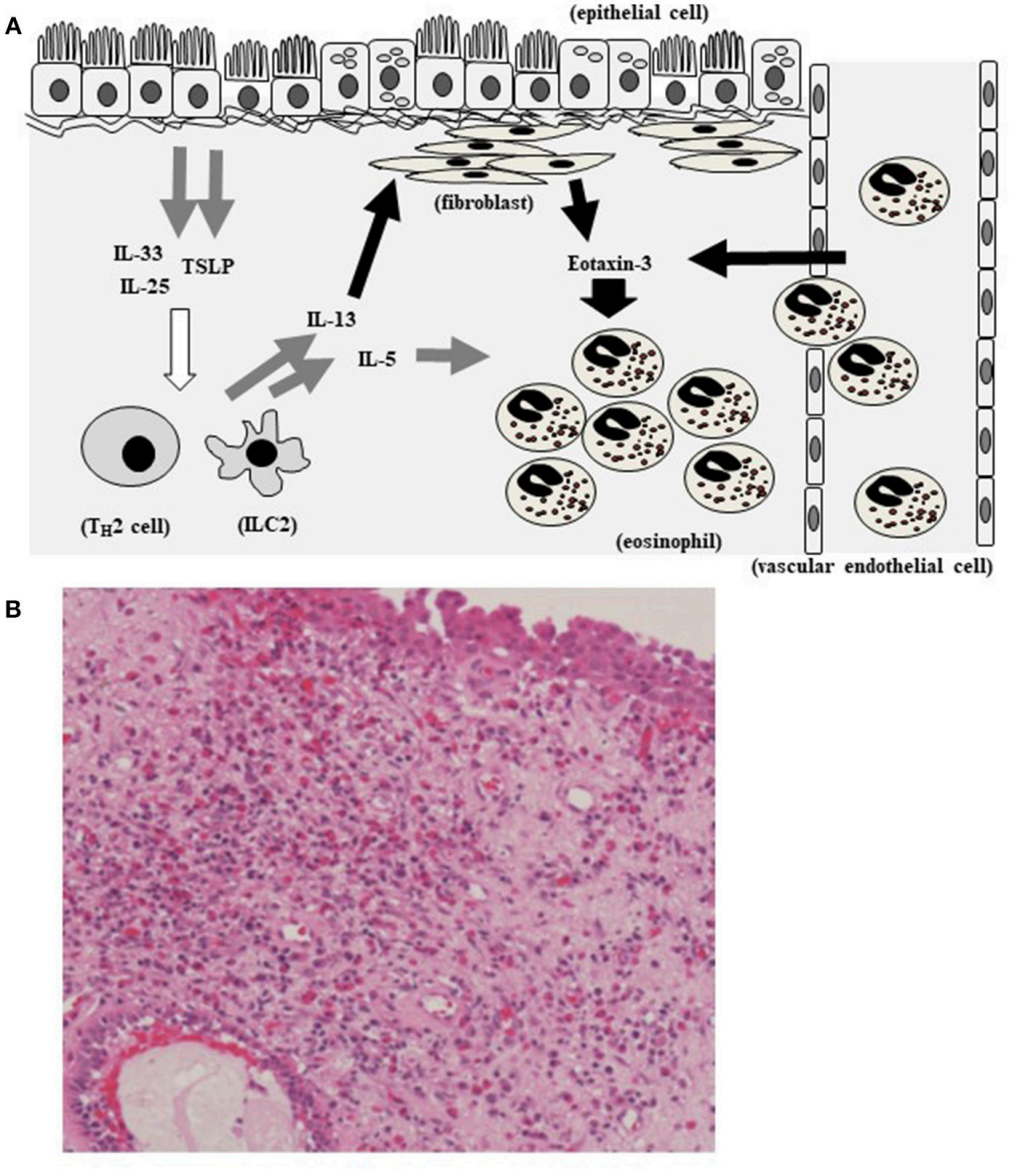
Hypothetical model showing eotaxin-3 and eosinophil infiltration in CRS. TSLP and IL-33 that are secreted by epithelial cells and act on type 2 ILCs and T_H_2 cells, inducing IL-5 and IL-13. IL-13 induces eotaxin-3 production from fibroblasts and epithelial cells. Eotaxin-3 induced the tissue infiltration of eosinophils **(A)**. Eosinophils infiltrate in the tissue of CRS (The section is stained with hematoxylin–eosin) **(B)**.

In this study, we found a significant positive correlation between the plasma level of eotaxin-3 and the mucosal eosinophil count. The level of eotaxin-3 in high-eosinophil mucosal infiltration group was significantly higher than that in low- group (*p* < 0.005). The nasal mucosal eosinophil counts, plasma IL-33 levels, and TSLP level were significantly higher in high-level eotaxin-3 group than those in low-level eotaxin-3 group. IL-13 strongly induced the secretion of eotaxin-3 from human nasal fibroblasts. Based on research data, TSLP and IL-33 that are secreted from epithelial cells and can induce type 2 ILCs and T_H_2 cells, and then the levels of IL-4, IL-5, and IL-13 increase. IL-13 induces eotaxin-3 production from fibroblasts and epithelial cells. Eotaxin-3 induced the tissue infiltration of eosinophils (Figure 6A). Thus, eotaxin-3 could function in pathology of mucosal eosinophil infiltration in CRSwNP. Bronchial epithelial cell injury leads to production of type 2 alarmins such as IL-33 and TSLP, also in asthma. Type 2 ILCs produce, in response to TSLP or IL-33, large amounts of the T_H_2 cytokines IL-5, IL-13 and, to a lesser extent, IL-4 (19, 20). Eotaxin-3 is the most efficient eotaxin to induce the migration or transmigration of eosinophils (21). The hematoxylin–eosin staining shows eosinophils infiltrate also in the tissue of CRS (Figure 6B).

Eosinophil-predominant disorders are closely related to allergic diseases, such as asthma, allergic rhinitis, atopic dermatitis, eosinophilic esophagitis. The eotaxin-3 expression was found optimal from the data set to define type 2 inflammation based on airway mucosal IL-13-driven gene expression and how this related to clinically accessible biomarkers for patients with mild to severe asthma and non-atopic healthy control subjects (22). Allergic subjects have a significant immediate response of nasal symptoms as well as a significant increase significantly raising levels of eotaxin-3 after nasal allergen challenge (23). The gene expression level for eotaxin-3 was higher in skin changes of atopic dermatitis patients (24). The serum eotaxin-3 level was significantly higher in patients with urticaria than in the healthy controls (25). The pathogenesis of eosinophilic esophagitis depends on local epithelial immune activation with production of eotaxin-3 and TSLP (26). On the microarray, 1,999 genes were differentially expressed between eosinophilic gastritis in children and the controls (*p* < 0.05), including significant upregulation of eotaxin-3 (27).

Precision medicine (28) is medical treatment that precisely classifies the patient's condition to select a suitable treatment method, personal medical care, and tailor-made medical treatment. Moreover, for CRS or allergic diseases, biomarkers, genetic factors, relationships with environmental factors, and phenotypes are often separated for therapy (29, 30). Defining endotypes can help clinicians predict disease prognosis, and select subjects suitable for a specific therapy for CRS (31). CRS is caused by dysregulated immunological responses inducing various mediators and inflammatory cells, including innate lymphoid cells, TSLP, and IL-33, which are mainly secreted by epithelial cells in response to external stimuli. They then act on type 2 ILCs and Th2 cells, inducing IL-4, IL-5, and IL-13. In this study, IL-33 and TSLP levels were significantly higher in the high-level eotaxin-3 group than those in the low-level eotaxin-3 group. We noted the significant positive correlation between the plasma levels of eotaxin-3 and IL-33 (*p* < 0.001) or TSLP (*p* < 0.005).

T_H_2 cytokines (IL-4 or IL-13) play important roles in CRS. IL-4 or IL-13 induce TSLP production by nasal fibroblasts (32). Local IgE and B cell activating factor (BAFF, BLyS) production are also signature characteristics of nasal polyps (33). IL-4 induces class switch recombination to IgE with the CD40 ligand, BAFF, and BLyS (34). IL-4 and IL-13 also down-regulate tissue plasminogen activator (t-PA) in epithelial cells while t-PA activates fibrinolysis cascades involve in fibrin deposition and dissolution (35). IL-13 stimulation of human nasal epithelial cells dominantly induced eotaxin-3 expression (36). We also found IL-13 significantly increased eotaxin-3 production by human nasal fibroblasts. The strong induction of eotaxin-3 from nasal tissue, high levels of eotaxin-3 in nasal polyp (36), and its expression of endothelial cells (15) could keep the plasma high levels of eotaxin-3 in the patients with ECRS. The nasal mucosal eosinophil count was significantly 2 times higher in plasma high-level eotaxin-3 group than in low-level eotaxin-3 group. The blood eosinophil count was 1.2 times higher in high-level eotaxin-3 group than in low-level eotaxin-3 group while there was no significant difference in the percentage or the number of blood eosinophil between the two groups. Increased eotaxin-3 levels in tissue could induce mucosal infiltration of eosinophils more strongly compared with the increase of blood eosinophils. And, the recurrence rate of high-level eotaxin-3 group was significantly 3 times higher than that of low-level eotaxin-3 group for 3 years after surgeries (*p* < 0.05). Dupilumab is a fully human monoclonal antibody against the IL-4 receptor α subunit, which inhibits signaling of IL-4 and IL-13. Of note, during this trial, the serum eotaxin-3 levels decreased and then nasal polyp scores began to decrease. On the other hand, the blood eosinophil count did little change in the group using dupilumab (37).

Human monoclonal antibodies are available or under clinical trials in allergic disease or refractory CRS. T_H_2 cytokines such as IL-4, IL-5, IL-13, epithelial-derived cytokines such as TSLP, IL-33, and acquired immunity markers including IgE could be candidates for molecular targets. In the randomized, double- blind, placebo-controlled study on the effects of humanized monoclonal anti-IgE antibody, omalizumab on NP and comorbid asthma (38), omalizumab reduced the total nasal endoscopic polyp score and demonstrated significant benefits for nasal and respiratory symptoms, such as nasal congestion, anterior rhinorrhea, loss of smelling sense, wheezing, and dyspnea. On the contrary, there was no reduction in inflammatory mediators in the treated group. Eosinophils, mast cells, and ILC2s mainly produce IL-5. The anti-IL-5 mAb, reslizumab reduced the polyp size, blood eosinophilic counts, and ECP concentration in nasal secretions (39). Responders had higher IL-5 levels in nasal secretions compared with non-responders. In the clinical trial of the anti-IL-5 mAb, mepolizumab, there was significant improvement in the nasal polyposis severity, VAS score, endoscopic nasal polyp score, and all individual VAS symptom scores in the mepolizumab compared with placebo groups (40). However, it remains unclear which biomarker can be used to select good responders to anti-IL-5 treatment. Dupilumab can inhibit signaling of IL-4 and IL-13 central to T_H_2-cell–mediated inflammation. Its clinical efficacy for T_H_2-cell–mediated diseases of asthma and atopic dermatitis has been confirmed and it also improved the nasal symptoms in perennial allergic rhinitis and comorbid asthma (41). A randomized clinical trial demonstrated a potential role of dupilumab in NP by significantly decreasing the nasal polyp score (*p* < 0.001) and improving in olfaction and CT scores (*p* < 0.001), as well as other clinical outcomes such as nasal symptoms and quality of life (37). Significant improvements with dupilumab were also observed for the 22-item sinonasal symptoms (*p* < 0.001). The serum eotaxin-3 levels decreased within 2 weeks, nasal polyp scores began to decrease at 4 weeks, and then sinonasal symptoms improved gradually. The serum levels of total IgE also decreased thereafter.

As CRS involves several mechanisms with high heterogeneity and different therapeutic responses, biomarkers and endotyping (42, 43) help to determine the optimal primary therapeutic modality, select good responder to a specific treatment, and predict treatment outcomes. In this study, the significant positive correlation between the plasma levels of eotaxin-3 and the mucosal eosinophil count support the ability of dupilumab to reduce nasal mucosal eosinophil infiltration against little change of blood eosinophil counts. Dupilumab can reduce the blood eotaxin-3 levels that are significantly higher in the high-risk group for recurrence after surgery. Also proton pump inhibitors can decrease eotaxin-3 expression in patients with CRSwNP (36). Eotaxin-3 levels could be one of the suitable plasma biological parameters for the efficacy of dupilumab or proton pump inhibitors in CRS. This may be a breakthrough in the treatment of recalcitrant CRS. Further studies including multivariate analysis are needed to investigate the potential use of monoclonal antibodies as adjunct therapy or with other medications or surgery.

## Ethics Statement

This study was carried out in accordance with the recommendations of Declaration of Helsinki with written informed consent from all subjects. All subjects gave written informed consent in accordance with the Declaration of Helsinki. The protocol was approved by the ethics committee of each institution including the general public through the Division of Otorhinolaryngology, Head and Neck Surgery, University of Fukui.

## Author Contributions

TY, HS, and YM: conception and design; TY, HS, YM, SU, TT, MS, YKat, TN, SF, YKaw, and SS: analysis and interpretation; TY, HS, and YM: drafting the manuscript for important intellectual content.

### Conflict of Interest Statement

The authors declare that the research was conducted in the absence of any commercial or financial relationships that could be construed as a potential conflict of interest.

## Abbreviations

HPF: high-power field
CRSwNP: chronic rhinosinusitis with nasal polyps
CCL: chemokine ligand
CRSsNP: chronic rhinosinusitis without nasal polyps
ECRS: Eosinophilic chronic rhinosinusitis
ESS: endoscopic sinus surgery
IL: interleukin
ROC: receiver operating characteristic
AUC: area under the curve
TSLP: Thymic stromal-derived lymphopoietin
T_H_2: T helper 2
ELISA: enzyme-linked immunosorbent assay
ILC2: innate lymphoid cells
BAFF: B cell activating factor
tPA: tissue plasminogen activator.
